# Aqua­chlorido{6,6′-dimeth­oxy-2,2′-[ethane-1,2-diylbis(nitrilo­dimethyl­idyne)]diphenolato-κ^2^ *O* ^1^,*N*,*N*′,*O* ^1′^}cobalt(III) monohydrate

**DOI:** 10.1107/S1600536809011167

**Published:** 2009-03-31

**Authors:** Jianxin Xing

**Affiliations:** a Department of Biology, Dezhou University, Dezhou 253023, People’s Republic of China

## Abstract

The title compound, [Co(C_18_H_18_N_2_O_4_)Cl(H_2_O)]·H_2_O, contains a distorted octa­hedral cobalt(III) complex with a 6,6′-dimeth­oxy-2,2′-[ethane-1,2-diylbis(nitrilo­dimethyl­idyne)]diphenolate ligand, a chloride and an aqua ligand, and also a disordered water solvent mol­ecule (half-occupancy). The Co^III^ ion is coordinated in an N_2_O_3_Cl manner. Weak O—H⋯O hydrogen bonds may help to stabilize the crystal packing.

## Related literature

For related literature, see: Aurangzeb *et al.* (1994 ▶); Hulme *et al.* (1997 ▶); Li *et al.* (2008 ▶); Fei & Fang (2008 ▶); Wang *et al.* (1979 ▶); Xia *et al.* (2007 ▶); Zhang & Janiak (2001 ▶).
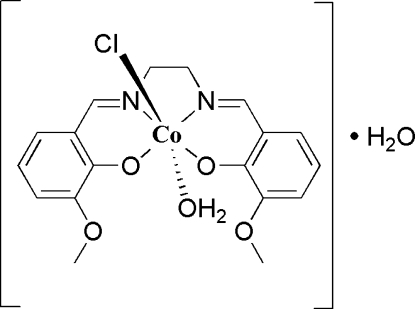


## Experimental

### 

#### Crystal data

[Co(C_18_H_18_N_2_O_4_)Cl(H_2_O)]·H_2_O
*M* *_r_* = 456.76
Trigonal, 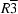

*a* = 26.490 (2) Å
*c* = 15.6234 (17) Å
*V* = 9494.5 (14) Å^3^
*Z* = 18
Mo *K*α radiation
μ = 0.98 mm^−1^
*T* = 293 K
0.15 × 0.13 × 0.09 mm

#### Data collection

Bruker APEXII CCD area-detector diffractometer
Absorption correction: multi-scan (*SADABS*; Sheldrick, 2003 ▶) *T*_min_ = 0.868, *T*_max_ = 0.917
13737 measured reflections
4116 independent reflections
2834 reflections with *I* > 2σ(*I*)
*R*_int_ = 0.062

#### Refinement

*R*[*F* ^2^ > 2σ(*F* ^2^)] = 0.074
*wR*(*F* ^2^) = 0.259
*S* = 1.03
4116 reflections
274 parameters
H-atom parameters constrained
Δρ_max_ = 1.55 e Å^−3^
Δρ_min_ = −1.03 e Å^−3^

### 

Data collection: *APEX2* (Bruker, 2004 ▶); cell refinement: *SAINT-Plus* (Bruker, 2001 ▶); data reduction: *SAINT-Plus*; program(s) used to solve structure: *SHELXS97* (Sheldrick, 2008 ▶); program(s) used to refine structure: *SHELXL97* (Sheldrick, 2008 ▶); molecular graphics: *XP* (Sheldrick, 1998 ▶); software used to prepare material for publication: *XP*.

## Supplementary Material

Crystal structure: contains datablocks global, I. DOI: 10.1107/S1600536809011167/hg2491sup1.cif

Structure factors: contains datablocks I. DOI: 10.1107/S1600536809011167/hg2491Isup2.hkl

Additional supplementary materials:  crystallographic information; 3D view; checkCIF report

## Figures and Tables

**Table 1:** Hydrogen-bond geometry (Å, °)

*D*—H⋯*A*	*D*—H	H⋯*A*	*D*⋯*A*	*D*—H⋯*A*
O7—H7*D*⋯O3^i^	0.86	2.44	2.883 (5)	113
O7—H7*D*⋯O5^i^	0.86	2.22	3.078 (5)	178
O7—H7*C*⋯O6^i^	0.84	2.58	3.033 (6)	115
O7—H7*C*⋯O4^i^	0.84	1.95	2.798 (5)	178
O2—H2*D*⋯O2^ii^	0.86	2.01	2.861 (9)	178
O2—H2*C*⋯O8^ii^	0.84	2.13	2.868 (19)	147
O2—H2*C*⋯O1^ii^	0.84	1.72	2.56 (3)	175
O8—H8*E*⋯O2^iii^	0.85	2.04	2.868 (19)	163
O8—H8*D*⋯Cl1	0.84	2.34	3.147 (12)	163
O1—H1*D*⋯Cl1	0.85	2.34	3.11 (3)	150

Symmetry codes: (i) 
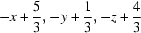
; (ii) 
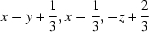
; (iii) 
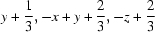
.

**Fig. 1.**
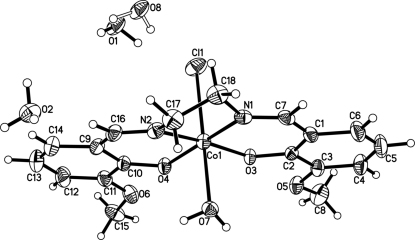
A view of complex (I), showing 30% probability displacement ellipsoids and the atom-numbering scheme. The solvate water molecule and all the H atoms have been omitted for clarity.

## supplementary crystallographic information

### Comment

The synthesis and structural investigation of Schiff base complexes have attracted much attention due to their interesting structures and wide potential applications. They play an important role in the development of coordination chemistry as well as inorganic biochemistry, catalysis, optical materials and so on (Aurangzeb *et al.*, 1994, Hulme *et al.*, 1997; Li *et al.*, 2008; Fei & Fang, 2008; Zhang & Janiak, 2001). Here, we report a new Schiff base cobalt complex based on the tetradentate Schiff base ligand 6,6'-dimethoxy-2,2'-(ethane-1,2-diyldiiminodimethylene)diphenol.

The molecular structure of title compound is shown in Fig. 1. The coordination sphere for the Co^III^ ion in the title complex is a distorted octahedron, in which four equational positions come from two N atoms, two O atoms of the Schiff base ligand, and the other two *trans* ones are occupied by one chloro ion and the O atom of water molecule. The Co—O and Co—N bond lengths are basically consistent with the corresponding distances in the similar cobalt tetradentate Schiff base complex bis[[µ-bis(salicylaldehyde)ethylenediimine]-dicobalt(III) dichloride chloroform solvate(Wang, *et al.*, 1979), while the Co—O (H_2_O) and the Co—Cl bond lengths are slightly longer than those found in the same complex. Additional, molecules are held together *via* intermolecular O—H···O and intramolecular O—H···Cl and O—H···O hydrogen bonds.

### Experimental

6,6'-dimethoxy-2,2'-(ethane-1,2-diyldiiminodimethylene)diphenol was synthesized according to a modified reported method (Xia, *et al.*, 2007). A mixture of CoCl_2_.6H_2_O (1 mmol, 237 mg), 6,6'-dimethoxy-2,2'-(ethane-1,2-diyldiiminodimethylene)diphenol (1 mmol, 326.4 mg) and 40 ml methanol was stirred for 30 min at 323 K, before it was filtered to remove the insolvable materials. Crystals suitable for X-ray diffraction analysis were obtained by slow evaparation at room temperature for three weeks with a yield about 40%.

### Refinement

All H atoms bonded to the C atoms were placed in geometrically calculated positions with C—H = 0.96 Å for methyl H atoms, C—H = 0.97 Å for methylene H atoms, C—H = 0.93 Å for aromatic H atoms and were refined isotropic with *U*_iso_(H) = 1.2*U*_eq_(C) or *U*_iso_(H) = 1.5*U*_eq_(C) of parent atom using a riding model. The H atoms of the disordered H_2_O were located from difference maps, in which the H_2B_ and H_2C_ were also disordered with the individual occupancy of 25%, and the O—H bond lengths were constrained to the value of 0.85 (1)Å with *U*_iso_(H) = 1.2*U*_eq_(O).

### Crystal data

**Table:**

[Co(C_18_H_18_N_2_O_4_)Cl(H_2_O)]·H_2_O	*D*_x_ = 1.438 Mg m^−^^3^*D*_m_ = 1.438 Mg m^−^^3^*D*_m_ measured by not measured
*M**_r_* = 456.76	Mo *K*α radiation, λ = 0.71073 Å
Trigonal, *R*3	Cell parameters from 5356 reflections
Hall symbol: -R 3	θ = 2.7–26.9°
*a* = 26.490 (2) Å	µ = 0.98 mm^−^^1^
*c* = 15.6234 (17) Å	*T* = 293 K
*V* = 9494.5 (14) Å^3^	Block, orange
*Z* = 18	0.15 × 0.13 × 0.09 mm
*F*(000) = 4248	

### Data collection

**Table:**

Bruker APEXII CCD area-detector diffractometer	4116 independent reflections
Radiation source: fine-focus sealed tube	2834 reflections with *I* > 2σ(*I*)
graphite	*R*_int_ = 0.062
φ and ω scans	θ_max_ = 26.2°, θ_min_ = 1.5°
Absorption correction: multi-scan (*SADABS*; Sheldrick, 2003)	*h* = −28→32
*T*_min_ = 0.868, *T*_max_ = 0.917	*k* = −32→25
13737 measured reflections	*l* = −19→12

### Refinement

**Table:**

Refinement on *F*^2^	Primary atom site location: structure-invariant direct methods
Least-squares matrix: full	Secondary atom site location: difference Fourier map
*R*[*F*^2^ > 2σ(*F*^2^)] = 0.074	Hydrogen site location: inferred from neighbouring sites
*wR*(*F*^2^) = 0.259	H-atom parameters constrained
*S* = 1.03	*w* = 1/[σ^2^(*F*_o_^2^) + (0.1835*P*)^2^] where *P* = (*F*_o_^2^ + 2*F*_c_^2^)/3
4116 reflections	(Δ/σ)_max_ = 0.001
274 parameters	Δρ_max_ = 1.55 e Å^−^^3^
0 restraints	Δρ_min_ = −1.03 e Å^−^^3^

### Special details

**Table:**

Geometry. All esds (except the esd in the dihedral angle between two l.s. planes) are estimated using the full covariance matrix. The cell esds are taken into account individually in the estimation of esds in distances, angles and torsion angles; correlations between esds in cell parameters are only used when they are defined by crystal symmetry. An approximate (isotropic) treatment of cell esds is used for estimating esds involving l.s. planes.
Refinement. Refinement of F^2^ against ALL reflections. The weighted R-factor wR and goodness of fit S are based on F^2^, conventional R-factors R are based on F, with F set to zero for negative F^2^. The threshold expression of F^2^ > 2sigma(F^2^) is used only for calculating R-factors(gt) etc. and is not relevant to the choice of reflections for refinement. R-factors based on F^2^ are statistically about twice as large as those based on F, and R- factors based on ALL data will be even larger.

### Fractional atomic coordinates and isotropic or equivalent isotropic displacement parameters (Å^2^)

**Table:**

	*x*	*y*	*z*	*U*_iso_*/*U*_eq_	Occ. (<1)
Co1	0.73792 (3)	0.12280 (3)	0.59262 (4)	0.0357 (3)	
Cl1	0.62851 (7)	0.08016 (8)	0.61355 (10)	0.0637 (5)	
O1	0.5945 (14)	0.144 (3)	0.478 (3)	0.057 (16)	0.15 (3)
H1C	0.6127	0.1636	0.4355	0.068*	0.15 (3)
H1D	0.6144	0.1305	0.5014	0.068*	0.15 (3)
O8	0.5817 (6)	0.1059 (11)	0.4435 (11)	0.058 (7)	0.35 (3)
H8E	0.5971	0.1406	0.4241	0.069*	0.35 (3)
H8D	0.5990	0.1068	0.4887	0.069*	0.35 (3)
O2	0.7381 (4)	0.2826 (4)	0.3172 (5)	0.055 (2)	0.50
H2C	0.7532	0.2773	0.2735	0.066*	0.25
H2D	0.7539	0.3193	0.3258	0.066*	0.50
H2B	0.7012	0.2675	0.3109	0.066*	0.25
O3	0.74870 (15)	0.07928 (14)	0.6768 (2)	0.0349 (8)	
O4	0.76146 (16)	0.18833 (15)	0.6619 (2)	0.0404 (8)	
O5	0.77398 (18)	0.04171 (17)	0.8092 (2)	0.0481 (9)	
O6	0.78604 (19)	0.25833 (17)	0.7866 (3)	0.0552 (10)	
O7	0.83443 (14)	0.15912 (15)	0.5522 (2)	0.0401 (8)	
H7C	0.8552	0.1539	0.5881	0.048*	
H7D	0.8499	0.1958	0.5434	0.048*	
N1	0.71890 (18)	0.06087 (19)	0.5088 (2)	0.0376 (9)	
N2	0.73614 (19)	0.1675 (2)	0.4961 (3)	0.0422 (10)	
C1	0.7312 (2)	−0.0054 (2)	0.5961 (3)	0.0417 (12)	
C2	0.7457 (2)	0.0273 (2)	0.6710 (3)	0.0352 (10)	
C3	0.7579 (3)	0.0050 (2)	0.7421 (3)	0.0429 (12)	
C4	0.7545 (3)	−0.0489 (3)	0.7403 (4)	0.0608 (17)	
H4	0.7633	−0.0628	0.7894	0.073*	
C5	0.7387 (4)	−0.0815 (3)	0.6683 (5)	0.074 (2)	
H5	0.7354	−0.1181	0.6685	0.088*	
C6	0.7280 (3)	−0.0612 (3)	0.5979 (5)	0.0592 (16)	
H6	0.7182	−0.0835	0.5482	0.071*	
C7	0.7192 (2)	0.0129 (2)	0.5214 (3)	0.0399 (11)	
H7	0.7102	−0.0118	0.4745	0.048*	
C8	0.7796 (3)	0.0185 (4)	0.8876 (4)	0.070 (2)	
H8A	0.7445	−0.0177	0.8988	0.104*	
H8B	0.7861	0.0456	0.9329	0.104*	
H8C	0.8120	0.0118	0.8844	0.104*	
C9	0.7448 (2)	0.2507 (3)	0.5713 (4)	0.0462 (12)	
C10	0.7583 (2)	0.2354 (2)	0.6479 (3)	0.0406 (12)	
C11	0.7708 (3)	0.2746 (2)	0.7164 (4)	0.0473 (13)	
C12	0.7685 (3)	0.3254 (3)	0.7061 (5)	0.0666 (18)	
H12	0.7766	0.3503	0.7525	0.080*	
C13	0.7547 (4)	0.3394 (3)	0.6299 (6)	0.079 (2)	
H13	0.7527	0.3733	0.6243	0.095*	
C14	0.7439 (3)	0.3038 (3)	0.5620 (5)	0.0635 (17)	
H14	0.7358	0.3140	0.5089	0.076*	
C15	0.8063 (3)	0.2980 (3)	0.8565 (4)	0.0628 (18)	
H15A	0.8427	0.3318	0.8415	0.094*	
H15B	0.8117	0.2795	0.9056	0.094*	
H15C	0.7781	0.3096	0.8695	0.094*	
C16	0.7376 (3)	0.2170 (3)	0.4987 (4)	0.0477 (13)	
H16	0.7334	0.2317	0.4468	0.057*	
C17	0.7351 (3)	0.1387 (3)	0.4155 (3)	0.0537 (15)	
H17A	0.7745	0.1508	0.3975	0.064*	
H17B	0.7166	0.1496	0.3712	0.064*	
C18	0.7020 (3)	0.0749 (3)	0.4289 (3)	0.0537 (14)	
H18A	0.6606	0.0613	0.4294	0.064*	
H18B	0.7099	0.0555	0.3824	0.064*	

### Atomic displacement parameters (Å^2^)

**Table:**

	*U*^11^	*U*^22^	*U*^33^	*U*^12^	*U*^13^	*U*^23^
Co1	0.0441 (5)	0.0379 (4)	0.0280 (4)	0.0226 (3)	−0.0021 (3)	−0.0013 (2)
Cl1	0.0478 (9)	0.0909 (12)	0.0596 (10)	0.0402 (9)	0.0133 (7)	0.0135 (8)
O1	0.053 (18)	0.08 (3)	0.05 (2)	0.04 (2)	0.001 (14)	0.02 (2)
O8	0.054 (8)	0.079 (15)	0.050 (9)	0.041 (9)	0.001 (6)	0.023 (9)
O2	0.059 (5)	0.064 (5)	0.038 (4)	0.028 (4)	−0.016 (3)	−0.006 (4)
O3	0.048 (2)	0.0316 (17)	0.0316 (16)	0.0247 (16)	−0.0040 (14)	−0.0019 (13)
O4	0.055 (2)	0.0358 (19)	0.0366 (18)	0.0271 (17)	−0.0075 (15)	−0.0045 (14)
O5	0.067 (3)	0.050 (2)	0.0366 (19)	0.036 (2)	−0.0030 (17)	0.0073 (16)
O6	0.070 (3)	0.041 (2)	0.052 (2)	0.025 (2)	−0.0006 (19)	−0.0114 (17)
O7	0.039 (2)	0.0397 (19)	0.0370 (19)	0.0165 (16)	−0.0023 (14)	0.0003 (14)
N1	0.035 (2)	0.044 (2)	0.031 (2)	0.0166 (19)	−0.0035 (16)	−0.0091 (17)
N2	0.043 (2)	0.052 (3)	0.033 (2)	0.025 (2)	−0.0031 (18)	0.0039 (18)
C1	0.040 (3)	0.033 (3)	0.051 (3)	0.018 (2)	−0.001 (2)	−0.009 (2)
C2	0.032 (2)	0.033 (2)	0.043 (3)	0.018 (2)	0.0029 (19)	0.0007 (19)
C3	0.052 (3)	0.040 (3)	0.041 (3)	0.027 (3)	0.004 (2)	0.008 (2)
C4	0.083 (5)	0.048 (4)	0.063 (4)	0.041 (4)	−0.004 (3)	0.008 (3)
C5	0.100 (6)	0.042 (4)	0.091 (6)	0.044 (4)	−0.014 (4)	−0.005 (3)
C6	0.072 (4)	0.038 (3)	0.070 (4)	0.029 (3)	−0.002 (3)	−0.013 (3)
C7	0.035 (3)	0.037 (3)	0.041 (3)	0.014 (2)	−0.004 (2)	−0.015 (2)
C8	0.096 (5)	0.088 (5)	0.038 (3)	0.056 (5)	0.000 (3)	0.018 (3)
C9	0.044 (3)	0.047 (3)	0.055 (3)	0.028 (3)	−0.001 (2)	0.007 (2)
C10	0.040 (3)	0.035 (3)	0.052 (3)	0.021 (2)	0.004 (2)	0.002 (2)
C11	0.047 (3)	0.038 (3)	0.057 (3)	0.021 (3)	0.002 (2)	−0.005 (2)
C12	0.082 (5)	0.046 (4)	0.080 (5)	0.039 (4)	−0.002 (4)	−0.011 (3)
C13	0.093 (6)	0.051 (4)	0.108 (6)	0.047 (4)	−0.005 (5)	0.003 (4)
C14	0.068 (4)	0.055 (4)	0.079 (4)	0.040 (3)	−0.003 (3)	0.008 (3)
C15	0.060 (4)	0.053 (4)	0.056 (4)	0.014 (3)	0.002 (3)	−0.023 (3)
C16	0.047 (3)	0.053 (3)	0.046 (3)	0.026 (3)	−0.005 (2)	0.013 (2)
C17	0.064 (4)	0.063 (4)	0.029 (3)	0.028 (3)	−0.005 (2)	−0.001 (2)
C18	0.062 (4)	0.066 (4)	0.033 (3)	0.032 (3)	−0.008 (2)	−0.009 (3)

### Geometric parameters (Å, °)

**Table:**

Co1—O3	1.863 (3)	C2—C3	1.370 (7)
Co1—O4	1.868 (3)	C3—C4	1.385 (8)
Co1—N2	1.932 (4)	C4—C5	1.350 (9)
Co1—N1	1.957 (4)	C4—H4	0.9300
Co1—O7	2.324 (3)	C5—C6	1.315 (10)
Co1—Cl1	2.5513 (17)	C5—H5	0.9300
O1—H1C	0.8381	C6—H6	0.9300
O1—H1D	0.8514	C7—H7	0.9300
O1—H8E	0.8596	C8—H8A	0.9600
O1—H8D	1.0649	C8—H8B	0.9600
O8—H1D	1.1965	C8—H8C	0.9600
O8—H8E	0.8530	C9—C10	1.367 (8)
O8—H8D	0.8360	C9—C16	1.396 (8)
O2—H2C	0.8374	C9—C14	1.426 (8)
O2—H2D	0.8561	C10—C11	1.411 (8)
O2—H2B	0.8563	C11—C12	1.385 (9)
O3—C2	1.342 (6)	C12—C13	1.350 (11)
O4—C10	1.309 (6)	C12—H12	0.9300
O5—C3	1.347 (7)	C13—C14	1.352 (10)
O5—C8	1.412 (7)	C13—H13	0.9300
O6—C11	1.314 (7)	C14—H14	0.9300
O6—C15	1.422 (7)	C15—H15A	0.9600
O7—H7C	0.8439	C15—H15B	0.9600
O7—H7D	0.8561	C15—H15C	0.9600
N1—C7	1.290 (7)	C16—H16	0.9300
N1—C18	1.436 (7)	C17—C18	1.480 (9)
N2—C16	1.294 (8)	C17—H17A	0.9700
N2—C17	1.465 (7)	C17—H17B	0.9700
C1—C7	1.362 (7)	C18—H18A	0.9700
C1—C2	1.391 (7)	C18—H18B	0.9700
C1—C6	1.438 (8)		
			
O3—Co1—O4	94.76 (14)	C6—C5—C4	119.7 (6)
O3—Co1—N2	171.32 (17)	C6—C5—H5	120.1
O4—Co1—N2	88.92 (18)	C4—C5—H5	120.1
O3—Co1—N1	90.48 (16)	C5—C6—C1	121.5 (6)
O4—Co1—N1	172.92 (17)	C5—C6—H6	119.3
N2—Co1—N1	85.24 (18)	C1—C6—H6	119.3
O3—Co1—O7	88.31 (14)	N1—C7—C1	126.8 (5)
O4—Co1—O7	89.17 (15)	N1—C7—H7	116.6
N2—Co1—O7	83.87 (16)	C1—C7—H7	116.6
N1—Co1—O7	86.23 (15)	O5—C8—H8A	109.5
O3—Co1—Cl1	97.31 (12)	O5—C8—H8B	109.5
O4—Co1—Cl1	96.58 (13)	H8A—C8—H8B	109.5
N2—Co1—Cl1	90.05 (14)	O5—C8—H8C	109.5
N1—Co1—Cl1	87.44 (13)	H8A—C8—H8C	109.5
O7—Co1—Cl1	171.56 (10)	H8B—C8—H8C	109.5
H1C—O1—H1D	108.1	C10—C9—C16	119.6 (5)
H1C—O1—H8E	39.0	C10—C9—C14	121.2 (6)
H1D—O1—H8E	105.8	C16—C9—C14	118.9 (5)
H1C—O1—H8D	113.4	O4—C10—C9	125.4 (5)
H1D—O1—H8D	33.5	O4—C10—C11	118.2 (5)
H8E—O1—H8D	89.9	C9—C10—C11	116.4 (5)
H1C—O8—H1D	65.3	O6—C11—C12	125.9 (6)
H1C—O8—H8E	16.5	O6—C11—C10	112.8 (5)
H1D—O8—H8E	81.6	C12—C11—C10	121.2 (6)
H1C—O8—H8D	91.9	C13—C12—C11	121.3 (6)
H1D—O8—H8D	26.9	C13—C12—H12	119.4
H8E—O8—H8D	108.1	C11—C12—H12	119.4
H2C—O2—H2D	108.4	C12—C13—C14	119.5 (6)
H2C—O2—H2B	111.0	C12—C13—H13	120.2
H2D—O2—H2B	110.1	C14—C13—H13	120.2
C2—O3—Co1	129.7 (3)	C13—C14—C9	120.3 (7)
C10—O4—Co1	129.6 (3)	C13—C14—H14	119.8
C3—O5—C8	115.2 (5)	C9—C14—H14	119.8
C11—O6—C15	117.5 (5)	O6—C15—H15A	109.5
Co1—O7—H7C	115.1	O6—C15—H15B	109.5
Co1—O7—H7D	108.4	H15A—C15—H15B	109.5
H7C—O7—H7D	108.2	O6—C15—H15C	109.5
C7—N1—C18	122.9 (4)	H15A—C15—H15C	109.5
C7—N1—Co1	126.7 (3)	H15B—C15—H15C	109.5
C18—N1—Co1	110.4 (4)	N2—C16—C9	126.8 (5)
C16—N2—C17	122.5 (5)	N2—C16—H16	116.6
C16—N2—Co1	126.9 (4)	C9—C16—H16	116.6
C17—N2—Co1	110.6 (4)	N2—C17—C18	108.7 (5)
C7—C1—C2	122.6 (5)	N2—C17—H17A	110.0
C7—C1—C6	118.7 (5)	C18—C17—H17A	110.0
C2—C1—C6	118.7 (5)	N2—C17—H17B	110.0
O3—C2—C3	118.8 (4)	C18—C17—H17B	110.0
O3—C2—C1	123.6 (5)	H17A—C17—H17B	108.3
C3—C2—C1	117.6 (5)	N1—C18—C17	109.6 (5)
O5—C3—C2	112.2 (4)	N1—C18—H18A	109.7
O5—C3—C4	126.4 (5)	C17—C18—H18A	109.7
C2—C3—C4	121.4 (5)	N1—C18—H18B	109.7
C5—C4—C3	121.0 (6)	C17—C18—H18B	109.7
C5—C4—H4	119.5	H18A—C18—H18B	108.2
C3—C4—H4	119.5		

### Hydrogen-bond geometry (Å, °)

**Table:**

*D*—H···*A*	*D*—H	H···*A*	*D*···*A*	*D*—H···*A*
O7—H7D···O3^i^	0.86	2.44	2.883 (5)	113
O7—H7D···O5^i^	0.86	2.22	3.078 (5)	178
O7—H7C···O6^i^	0.84	2.58	3.033 (6)	115
O7—H7C···O4^i^	0.84	1.95	2.798 (5)	178
O2—H2D···O2^ii^	0.86	2.01	2.861 (9)	178
O2—H2C···O8^ii^	0.84	2.13	2.868 (19)	147
O2—H2C···O1^ii^	0.84	1.72	2.56 (3)	175
O8—H8E···O2^iii^	0.85	2.04	2.868 (19)	163
O8—H8D···Cl1	0.84	2.34	3.147 (12)	163
O1—H1D···Cl1	0.85	2.34	3.11 (3)	150

Symmetry codes: (i) −*x*+5/3, −*y*+1/3, −*z*+4/3; (ii) *x*−*y*+1/3, *x*−1/3, −*z*+2/3; (iii) *y*+1/3, −*x*+*y*+2/3, −*z*+2/3.
